# Atypical Mycobacterial Tenosynovitis in the Setting of Adalimumab Use

**DOI:** 10.7759/cureus.18952

**Published:** 2021-10-21

**Authors:** Jay Patel, Nilmarie Guzman, Kurt Wukitsch

**Affiliations:** 1 Internal Medicine, Orange Park Medical Center, Orange Park, USA

**Keywords:** psoriasis, musculoskeletal, tenosynovitis, adalimumab, nontuberculous mycobacterium

## Abstract

Tumor necrosis factor-alpha (TNF-α) inhibitors indicated in the management of psoriasis, rheumatoid arthritis, ulcerative colitis, Crohn’s disease, and other autoimmune diseases have been associated with the development of mycobacterial and other opportunistic infections. The majority of mycobacterial infections diagnosed in patients taking TNF-α inhibitors are secondary to *Mycobacterium tuberculosis*. Atypical mycobacteria have also been identified in this patient population, most commonly manifested by pulmonary or disseminated infections. Extra-pulmonary manifestations such as bone and joint infections are rare. We describe a case of atypical mycobacterial tenosynovitis in the setting of adalimumab use in a patient with psoriasis. This is a rarely reported complication that one should be aware of when prescribing these medications.

## Introduction

Non-tuberculous mycobacteria (NTM) are ubiquitous species of organisms frequently present within reservoirs of water, soil, and animals [1]. NTM infections in immunocompetent individuals are quite rare, with reported cases most commonly occurring in the setting of risk factors such as environmental exposures, surgical site infections, and contaminated inoculations [2]. When these infections do occur, they most often present with pulmonary pathology. Other common infections encountered are lymphadenitis (young children) and disseminated disease in immunocompromised patients. Extra-pulmonary disease is rarely seen in immunocompetent patients [3].

Musculoskeletal (MSK) involvement is quite rare in presentation in comparison to pulmonary disease. When infection does involve the MSK system, hand and wrist involvement are most often reported. This has been hypothesized due to the rich synovial fluid environment present in these regions [2,4]. Of these patients, tenosynovitis is less common than arthritis or osteomyelitis [5]. Patients often present with insidious, slow, progressive entrapment of the tendons, which over time leads to worsening discomfort and decreased range of motion. Due to the gradual onset, symptoms tend to be quite mild at the onset, so patients tend to be quite advanced in their disease on presentation [6]. 

Tumor necrosis factor-alpha (TNF-α) inhibitors are often utilized in the management of rheumatoid arthritis, inflammatory bowel disease, and other autoimmune inflammatory conditions. Granulomatous infections, the most notable activation of latent tuberculosis, are a known complication of these medications through their immunosuppressive effects. However, their role in the development of NTM infections is not well-established, with extra-pulmonary involvement of tenosynovitis being a rarely reported adverse effect [7].

## Case presentation

**Figure 1:**
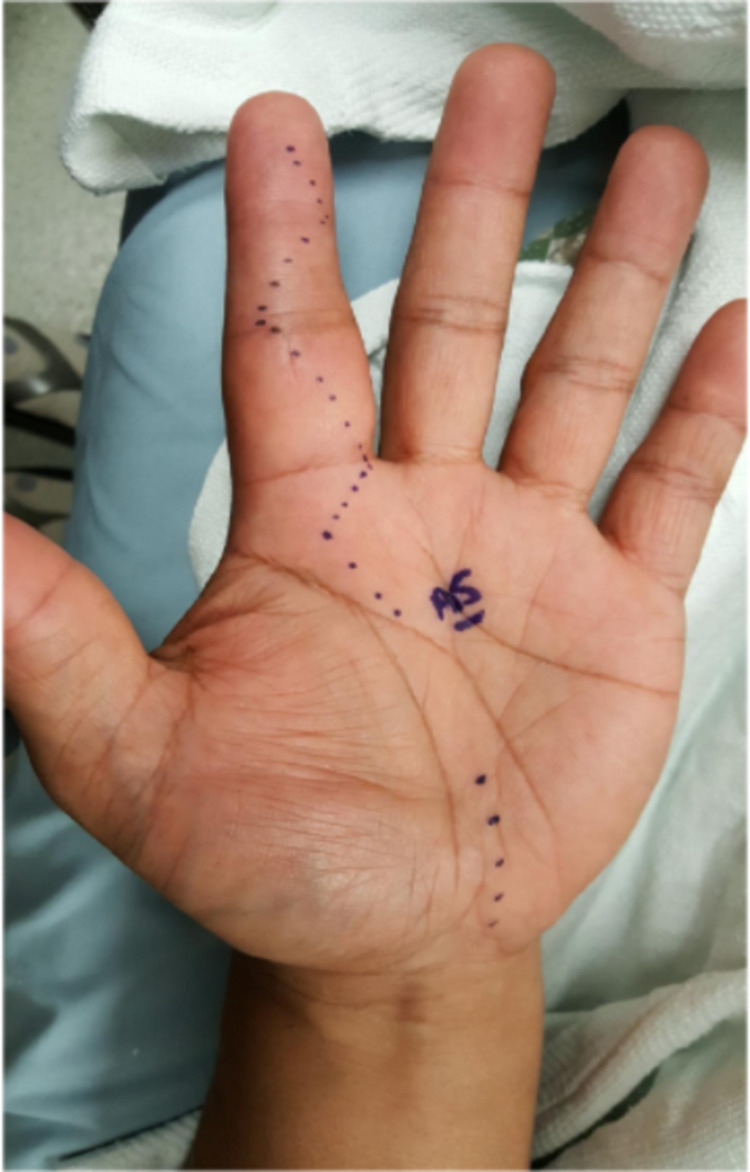
Left index finger with PIP swelling and erythema. PIP: proximal interphalangeal

A 60-year-old East-Asian male with a history of hypertension and psoriasis presented to his outpatient provider complaining of left index finger pain and swelling for three weeks. The swelling developed two days after getting a cooked eggshell wedged under his fingernail. He denied fevers, chills, discharge from his finger, or trauma. The patient had otherwise been in normal health and stable on chronic adalimumab therapy for over five years. He had a history of having taken the bacille Calmette-Guérin (BCG) vaccine and per chart had annual chest x-rays did which were unremarkable, the most recent being within six months of symptom onset. Physical examination revealed swelling of the left index finger extending from the proximal interphalangeal (PIP) joint to the metacarpophalangeal joint with tenderness along the ventral aspect, with no superficial skin breakdown noted (Figure 1). The pain was provoked with PIP extension and there was limited active and passive flexion of the digit. Initial labs showed an elevated erythrocyte sedimentation rate of 40mm/h and an elevated urate level of 8.4mg/dl. Due to the patient's family history of gout and presentation, a preliminary clinical diagnosis of gout was made by his prior provider, and the patient was started on gout therapy (unknown medication and dosage). Despite adjustments to dosages and medications for about a month, the patient would have worsening swelling in his finger.

**Figure 2:**
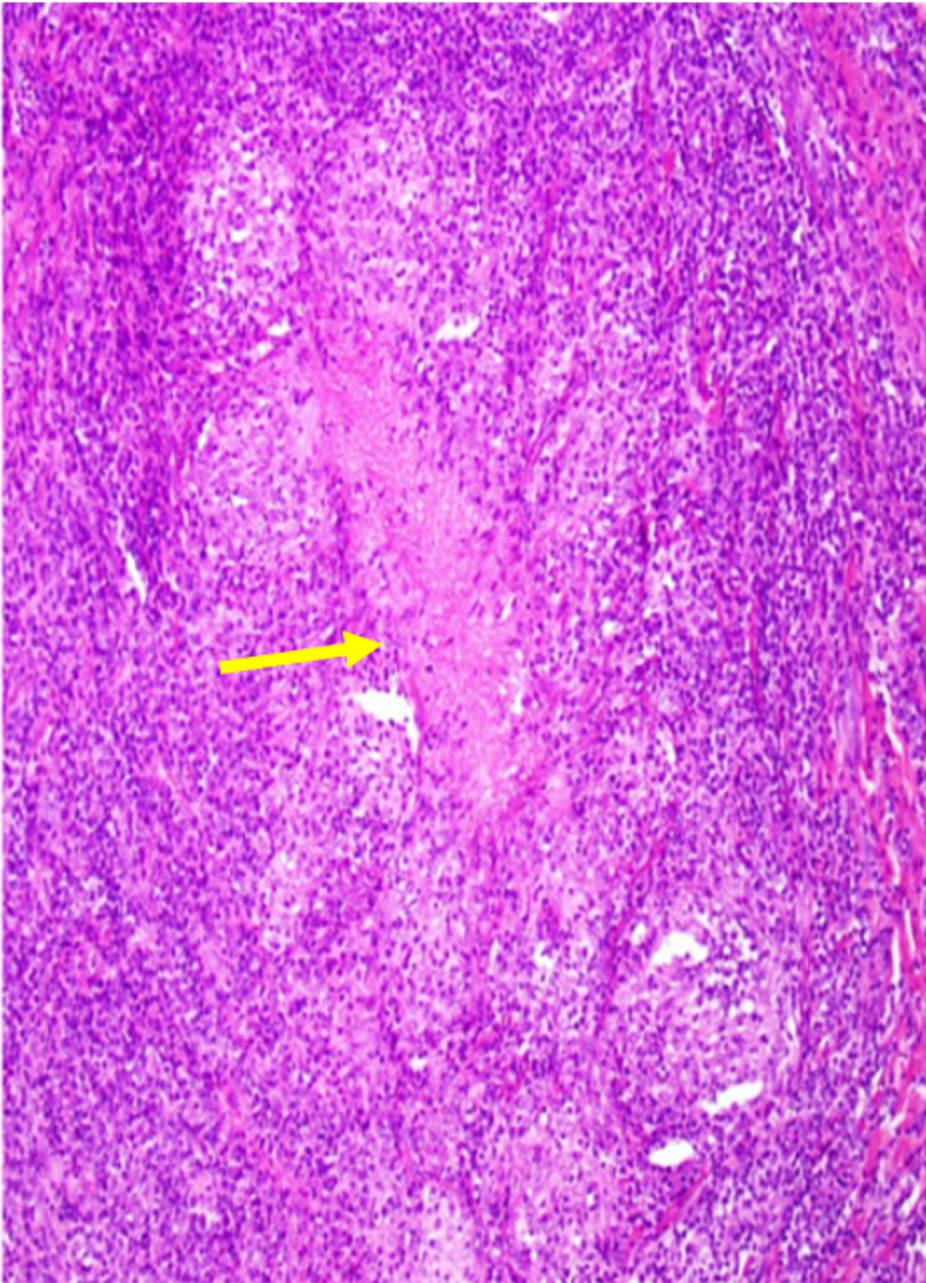
Necrotizing granuloma with epithelioid histiocytes surrounding central necrotic region. 10x magnification.

The patient was referred to an orthopedic surgeon and subsequent magnetic resonance imaging (MRI) showed possible flexor tendon tear of the left index finger. He underwent tenosynovectomy with the placement of a Hunter rod for stability. Tissue was sent for cultures and histopathology. Pathology showed necrotizing granulomas and cultures showed growth of *Mycobacterium avium* complex, resulting within three to four weeks of collection (Figure 2). It was at this point the patient transitioned care to our practice.

He was initiated on rifabutin, ethambutol, and clarithromycin (REC). Within a few weeks of therapy initiation, the patient would be noted to have an abscess on the palmar side of the same hand. Wound cultures again grew *Mycobacterium avium*. The Hunter rod was removed due to concerns for biofilm formation. The patient’s risk factor for the development of this infection was suspected to be the use of chronic adalimumab. Hence, after multidisciplinary provider discussion with the patient, adalimumab was discontinued. The patient was transitioned to apremilast 30mg within two to three weeks due to lower inherent risk for infection. The patient would be continued on antimycobacterial therapy for a minimum of 12 months and tolerated treatment well with gradual resolution of symptoms.

## Discussion

Non-tuberculous mycobacteria (NTM) make up a large group of environmental organisms found in soil and water that are well known to cause severe lung and disseminated disease in immunocompromised patients [2]. Their ability to make biofilms make them resistant to multiple antibiotics. They also have underlying hydrophobicity and have been found to be resistant to acidic and high temperature environments. Overall, over 150 species of mycobacterium have been identified, with increasing rates attributed to improvement in culture and differentiation techniques. NTM are typically classified according to their growth rate - fast versus slow. *Mycobacterium abscessus*, *Mycobacterium fortuitum*, *Mycobacterium chelonae* are known to be fast growers. The slow growers include *Mycobacterium avium* complex (MAC), *Mycobacterium kansasii*, *Mycobacterium xenopi*, and *Mycobacterium simiae* [1]. 

NTM infection is overall uncommon in immunocompetent hosts, with respiratory infection being the most common, with reported rates over 90% [2,4]. Underlying diseases such as emphysema, bronchiectasis, prior tuberculosis infection, rheumatoid arthritis, cystic fibrosis, and other chronic pulmonary conditions can predispose patients to NTM disease. Other reported extra-pulmonary infections include disseminated disease in immunocompromised patients, skin and soft tissue infections, and occasionally lymphadenitis [7]. It is not clear what underlying immunologic mechanisms may be contributing to predisposition. Some studies suggest problems in the production pathways of interleukin-12 or interferon-γ may be contributing to disease progression [8]. 

The incidence of NTM has been reported to be increasing in the past few decades; however, exact numbers are still not established as NTM infections are not required to be reported for public health purposes [9]. Due to overall lack of awareness and potential slow, gradual course, NTM tends to be underdiagnosed. Imaging findings can be unimpressive during both early and late disease course, resulting in delayed diagnosis and treatment [6].

Overall musculoskeletal involvement is quite rare, with one retrospective study of over 1000 patients reporting only around 3% of patients infected with NTM. Of these, only around a quarter had the presence of tenosynovitis [5]. When the hands are involved, tenosynovitis is the most common presentation of mycobacterial hand infection. However, in regards to NTM, hand infections are not that common, with *Mycobacterium marinum* being the most common organism [10]. When infections do occur, they often occur in the setting of underlying risk factors such as prior trauma, steroid infections, surgical procedures, or potentially even surgical inoculation [2, 11]. 

The hallmark of NTM tenosynovitis is the insidious and gradual onset of the disease, with progressive entrapment of the tendon [11]. Infection rarely involves the underlying bony structures or muscles, usually without tendon tear and preservation of the joint spaces [12]. Patients often present with pain, decreased range of motion, swelling, and possible drainage or palpable mass. 

In regards to diagnosis, early findings of soft tissue swelling and osteopenia of the joints are non-specific, with sometimes even imaging late in presentation being unimpressive [6,9]. The standard remains biopsy and culture of the synovium [9,11]. However, definitive diagnosis still remains difficult, with reports of diagnostic yield of acid-fast bacilli (AFB) staining being less than 60% and as low as 0% [9]. Identification of granulomas and especially rice bodies, which are small intra-articular bodies resembling small grains of white rice, are seen as crucial for diagnosis [11]. 

TNF-α is associated with granuloma maintenance and breakdown, and therefore medications that inhibit TNF-α may lead to an increased risk of granulomatous infections such as *Mycobacterium avium*. A data analysis study by Winthrop et al. evaluated the presence of NTM infections among patients using various TNF-α inhibitors such as infliximab, etanercept, and adalimumab. Among reports of over 200 NTM infections, adalimumab was the least reported, with concurrent bone or joint infection being even rarer [7].

## Conclusions

Muskuloskeletal involvement in NTM infections is overall rare and atypical. This case illustrates the increased risk for NTM infections in the setting of TNF-α inhibitors such as adalimumab. Early recognition may reduce the time before proper treatment is initiated. Concurrent co-infection should always be considered in patients on immunosuppressive therapy, with emphasis on adalimumab. NTM infection should be on the differential for patients presenting with possible infectious etiology in the setting of TNF-α inhibitor use.
